# Case Report: Ascending Aortic Pseudo-Aneurysm Following Ventricular Septal Defect Repair in a 4-Year-Old Girl

**DOI:** 10.3389/fped.2021.576527

**Published:** 2021-02-15

**Authors:** Xinya Li, Hong Zhou, Rui Zhang, Jing Zhao, Tian Li, Yu Zhang, Jianjun Ge

**Affiliations:** ^1^Department of Cardiovascular Surgery, The First Affiliated Hospital, University of Science and Technology of China, Hefei, China; ^2^Department of One-Day Ward, The First Affiliated Hospital, University of Science and Technology of China, Hefei, China; ^3^Department of Medical Imaging, The First Affiliated Hospital, University of Science and Technology of China, Hefei, China; ^4^School of Basic Medicine, Fourth Military Medical University, Xi'an, China; ^5^Xinchang Hospital Affiliated to Wenzhou Medical University, Shaoxing, China

**Keywords:** pseudo-aneurysm, ventricular septal defect, ascending aorta, cardiac surgery, cardiopulmoanry bypass

## Abstract

Pseudo-aneurysm is a fatal disease, and the main cause of death is massive hemorrhage secondary to the rupture of the aneurysm. This case report aims to evaluate the effects of pseudo-aneurysm excision procedure on the disease. A 4-year-old girl was readmitted on the 20th day after ventricular septal defect (VSD) closure procedure with a high fever of 40°C; aortic pseudo-aneurysm was suspected based on a spherical cystic echo (82 × 76 mm) of the ascending aorta which was detected by ultrasonic cardiogram, and the diagnosis was confirmed by an aortic computed tomograph angiography (CTA) examination and intraoperative findings. Treatment included emergency pseudo-aneurysm excision procedure and antibiotic therapy. The aortic pseudo-aneurysm was surgically removed under deep hypothermia and circulatory arrest. Antibiotics were applied according to the bacterial culture results. The pseudo-aneurysm was excised successfully, and the patient achieved a good recovery. Our case suggests that the postoperative ascending aortic pseudo-aneurysm was probably due to inappropriate purse-string suture and/or local or systematic infection, so extra precautions should be taken to avoid this life-threatening complication.

## Introduction

The ascending aortic pseudo-aneurysm (AAPA) is usually followed by aortic surgery in young patients. It is rare but carries a high risk of mortality (1). We presented a case with AAPA following ventricular septal defect (VSD) repair. Informed consent was obtained from patient's legal guardian for using patient's clinical information for submitting and publication.

## Case

A 4-year-old girl was admitted with a 3-year history of a cardiac murmur, and the admission weight was 17 kg. A 3/6 degree of systolic murmur at the third left intercostal space was detected by physical examination, and a perimembranous VSD of 6 mm was revealed by echocardiogram. The girl underwent VSD closure with inferior partial median sternotomy under cardiopulmonary bypass (CPB) at day 4 of hospitalization, and the VSD was closed with three interrupted horizontal mattress sutures. The operation was successful, and she was discharged at postoperative day 10.

**Figure 1:**
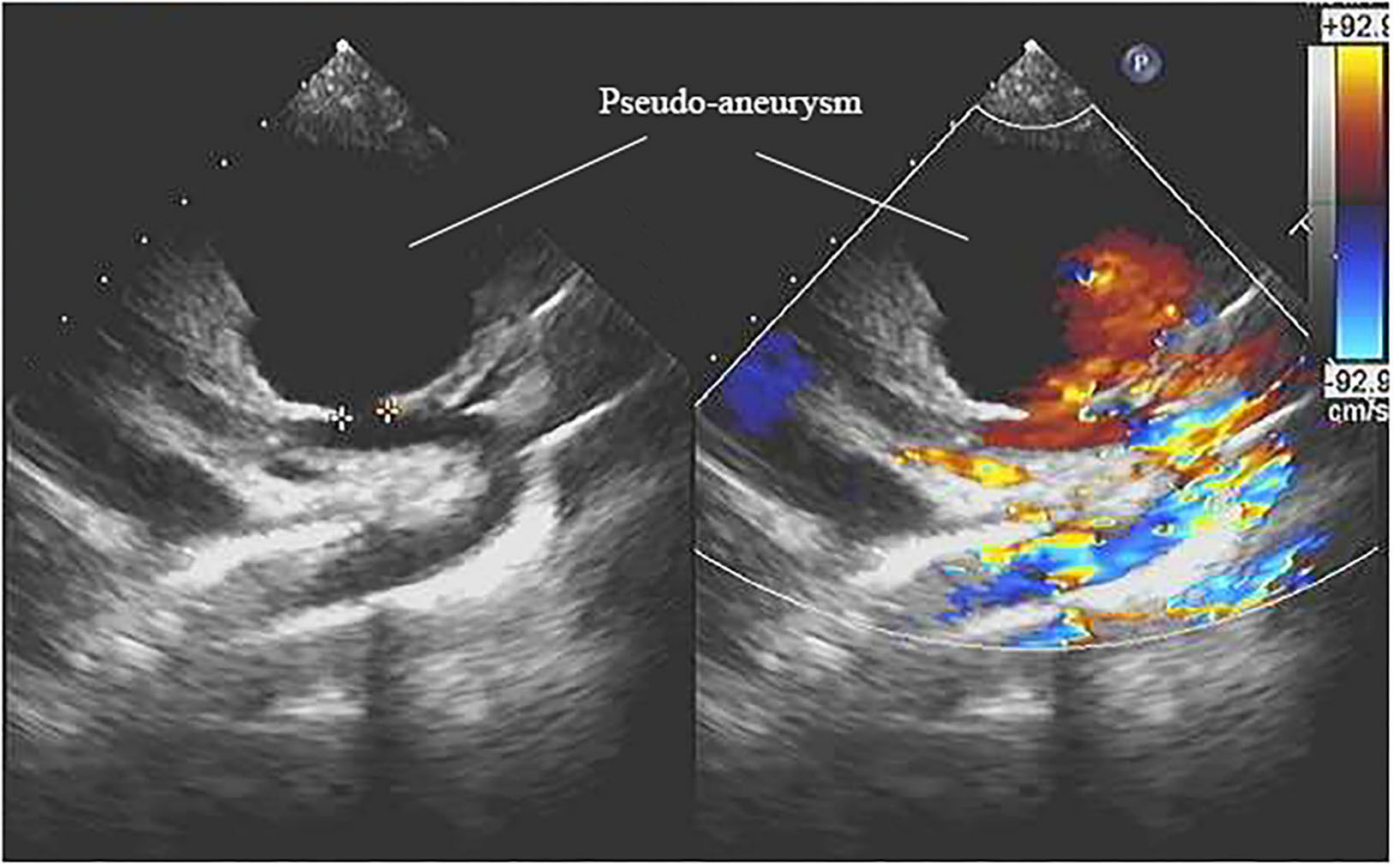
An echocardiography view represents the aortic pseudo-aneurysm.

**Figure 2:**
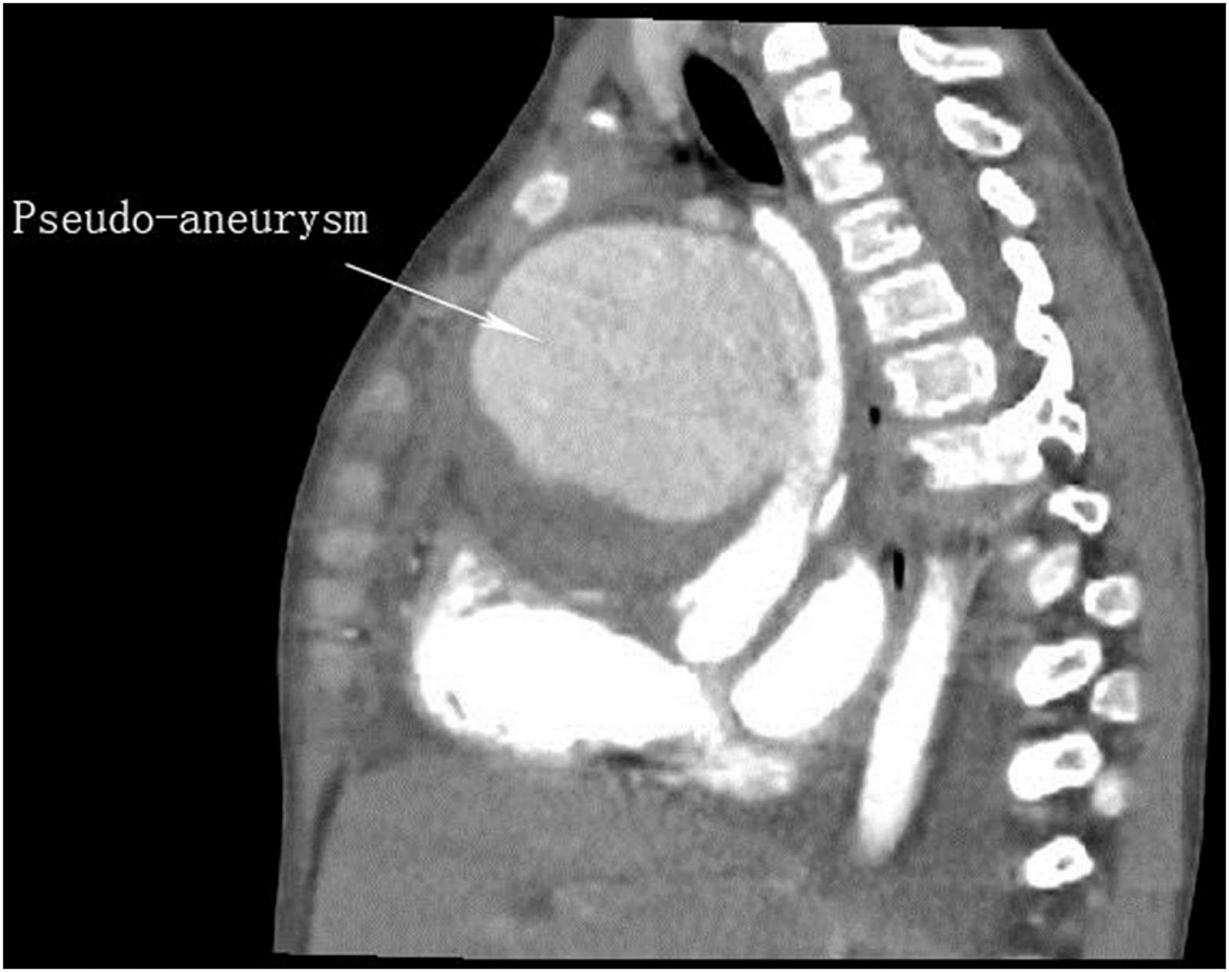
Sagittal view of contrast enhancement CT image shows a large pseudo-aneurysm is bound up with the ascending aorta.

**Figure 3:**
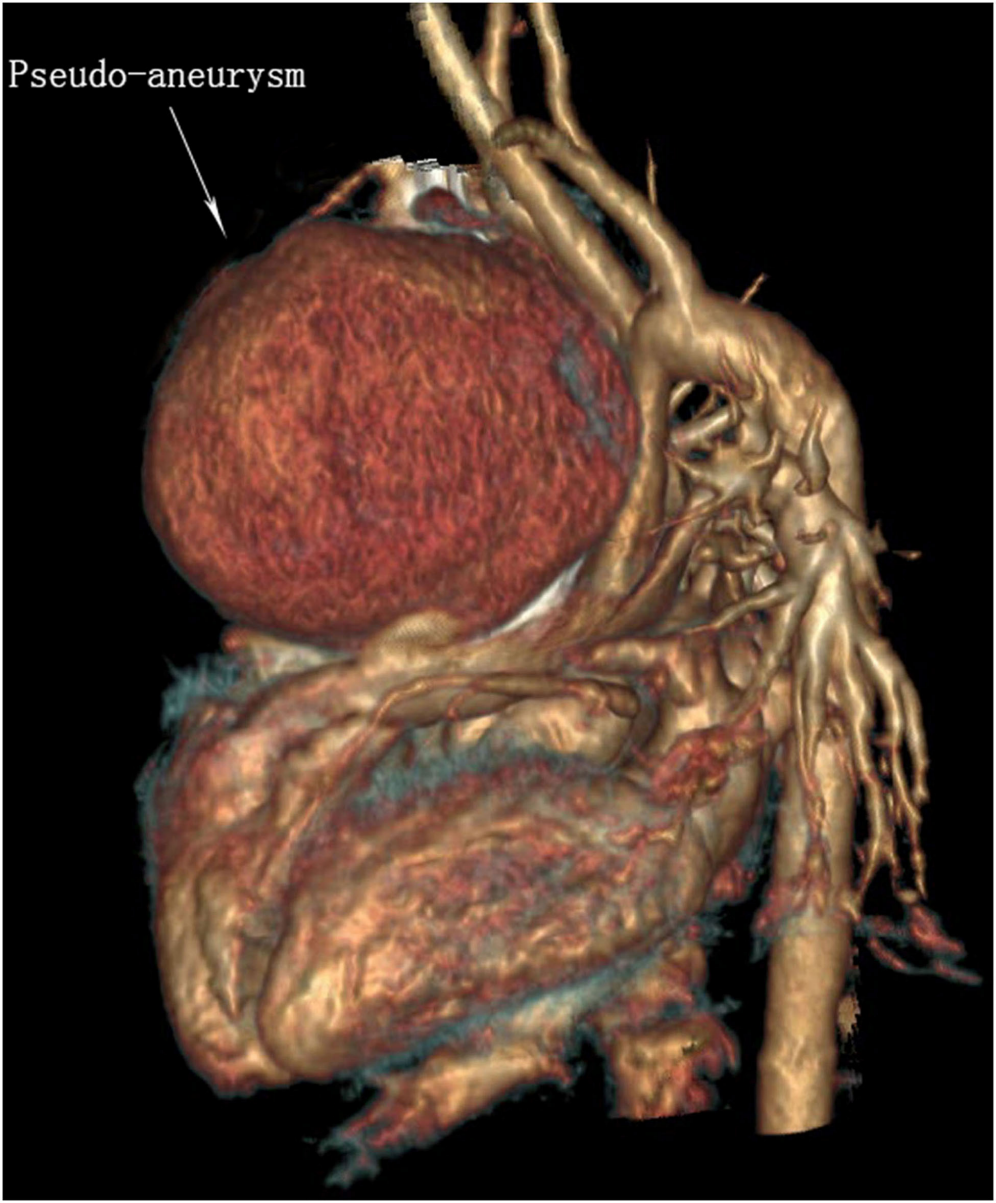
3D reconstruction of sagittal view of contrast enhancement CT image suggests a large pseudo-aneurysm is bound up with the ascending aorta.

She was readmitted at 20 days after the first surgery due to a high fever of 40°C at postoperative day 18 with no obvious causative factors and without characteristics of coughing, wheezing, retching, vomiting, or cardiac murmur. The girl showed a slight poor physical development and with no family history of genetic diseases and dysmorphism. She had received antibiotic therapy for 1 day before readmission at the local hospital. Laboratory examination showed that the white blood cell (WBC) count was 5.94 × 10^9^/L, and the percentage of neutrophils was 81.8%. Blood culture showed positive for *Staphylococcus aureus*. The electrocardiograph showed frequently occurring ventricular premature beat, and ultrasonic cardiogram (UCG) showed a spherical cystic echo (82 × 76 mm) at the anterolateral aspect of the ascending aorta (Figure 1). Then an emergent computed tomography angiography (CTA) was done which revealed an abnormal 86 × 78 mm echo located at the anterolateral aspect of the ascending aorta with a direct communication to the aorta, and the communicating hole was 8.9 mm in diameter (Figures 2, 3).

**Figure 4:**
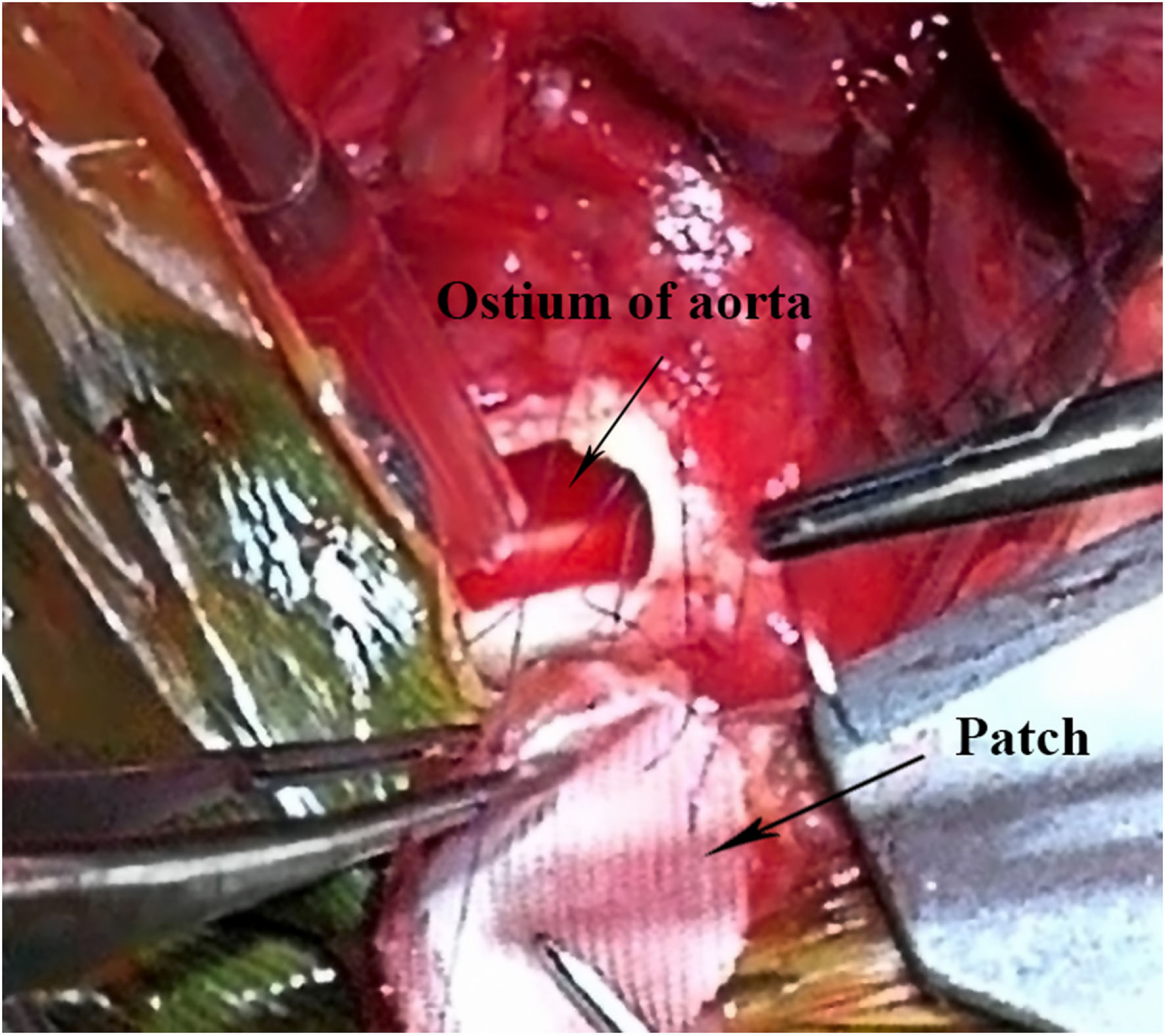
Intraoperative image demonstrates the ostium of aorta and the double layer patch used to repair the ostium.

**Figure 5:**
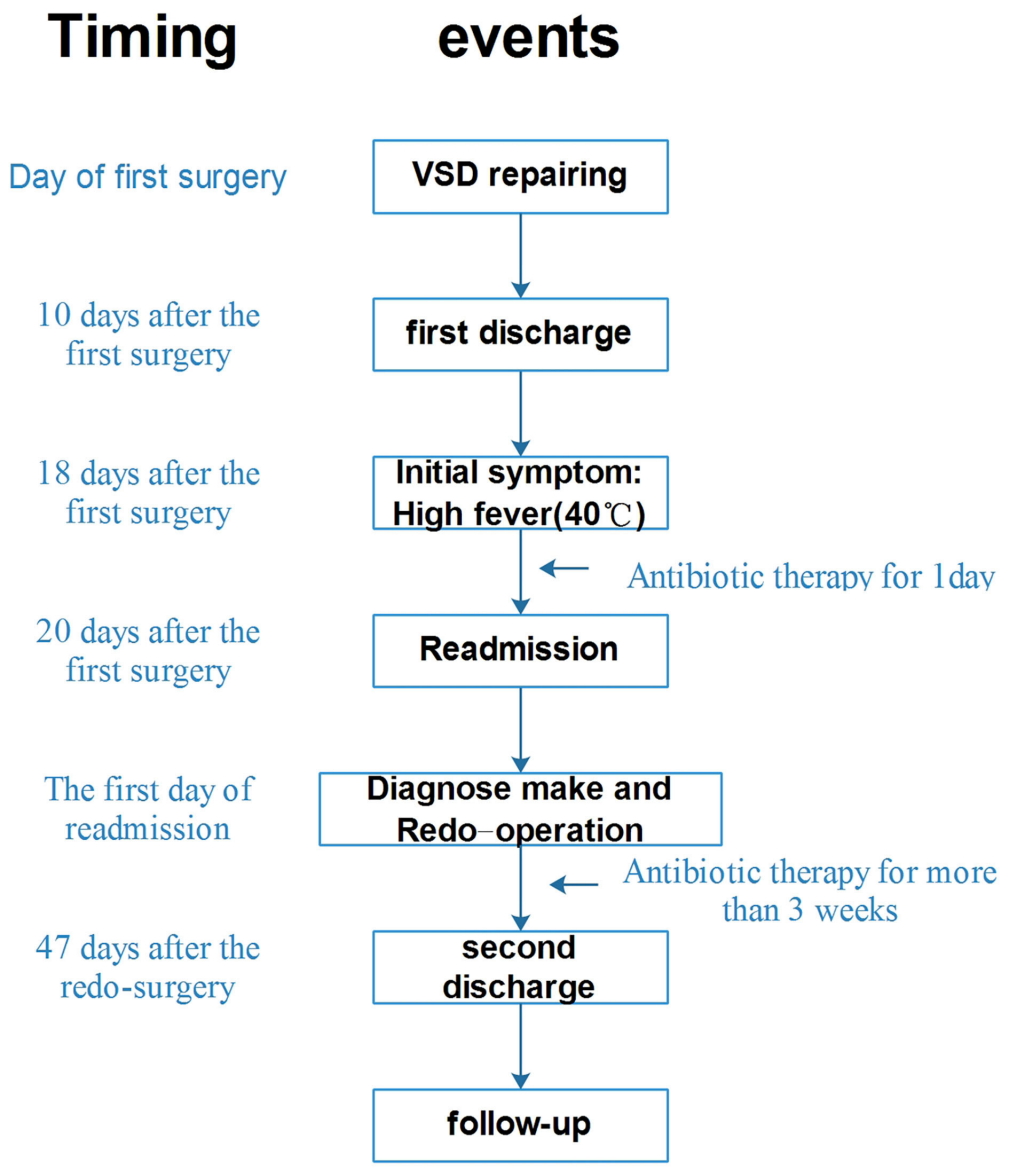
A flow diagram shows the overall therapeutic process.

An emergency operation was performed under deep hypothermia (20°C) with CPB. In detail, the right femoral artery and vein were cannulated using 14# arterial cannula and 19# venous cannula, respectively. Then median sternotomy was immediately performed after the CPB started. We found the pseudo-aneurysm at the anterior mediastinum wrapped with amounts of inflammatory scar and thymus tissue. Because of the giant pseudo-aneurysm and severe surrounding tissue adhesions, there was no sufficient space to place the aortic cross-clamp and cardioplegic cannula needle. Then the patient was rapidly cooled to 20°C, and the heart was arrested, induced by hypothermia, while an ice hat was used to implement brain local mild hypothermia therapy. After the patient was placed in a head-down position, we incised the pseudo-aneurysm rapidly and found an ostium (8 × 9 mm in diameter) in the anterior wall of ascending aorta where the purse-string suture was made during the previous surgery. Cardioplegia solution (St. Thomas solution, 30 ml/kg) was infused directly via the coronary ostia to achieve cardiac arrest. A piece of appropriate size of aneurysmal wall was harvested and soaked in iodophor for 2 min then overlaid with Dacron patch for closing the ostium with continuous suture of 4–0 prolene (Figure 4). After de-airing, the ostium was closed thoroughly, CPB was resumed, and the patient was warmed. After recovery to normal sinus rhythm, CPB was terminated, and the femoral cannulae were removed. The CPB time was 173 min, and circulatory arrest time was 15 min. After being weaned from CPB, the girl was closed up and returned to pediatric ICU for further therapy. Vancomycin and penicillin were administered intravenously for 10 days according to the results of drug sensitivity test, and then the oral antibiotics were administered until the day of discharge. The girl gradually recovered to asymptomatic condition and was discharged at 47 days after the secondary surgery. We followed up the girl for 2 years; the girl had a weight gain of 5 kg and good appetite that showed she is in good nutritional and well-developmental status. She received UCG and chest radiography examinations during the periodic outpatient reexaminations, and no abnormality was detected. The timeline of the overall therapeutic process is shown as a flow diagram in Figure 5.

## Discussion

We presented a rare case of a child with AAPA at early stage after cardiac surgery. The girl had no specific symptom except a high fever, and we failed to detect the retrosternal hematoma until the imaging examination was done. Pseudo-aneurysm is rare and associated with high mortality risk (2). In the present case, we performed the emergency surgery immediately after a definitive diagnosis. The pseudo-aneurysm was removed, and the dehiscence of the aorta was repaired successfully under deep hypothermic circulatory arrest. After that, she gradually recovered to asymptomatic condition and was discharged at postoperative day 47.

Pseudo-aneurysm is always fatal because of the serious bleeding complications. Morbidity of pseudo-aneurysm is <0.5% in all kinds of cardiac surgeries, and it usually occurs at the injured sites of the aorta, such as the aortic cannulation and suture sites. The possible reasons for the development of pseudo-aneurysm include aortic diseases, poor suture technique, perioperative infection, and biogel, all of which could make the aortic wall more fragile. In the present case, the positive blood culture result also suggested the pseudo-aneurysm was attributed to infection.

Regardless of size and site of the pseudo-aneurysm, surgical treatment is necessary once the diagnosis is definite. The mortality of surgical pseudo-aneurysm treatment ranges from 29 to 46%, and the main cause of death is massive hemorrhage secondary to the rupture during sternal reentry, especially when the pseudo-aneurysm is larger than 55 mm (3). The keystone of surgery is to prevent the hemorrhage so that median sternotomy under femorofemoral bypass and hypothermic circulatory arrest are recommended. In the present case, hypothermic circulatory arrest technology was applied, and we reentered the chest safely.

Similar to other studies, some cases adopted femoral artery cannulation to institute CPB (4), while others adopt the right axillary artery, subclavian artery, or ascending aorta (1, 3, 5). These adoptions of unconventional cannulation sites were due to retrosternal adhesion and approximation of the aorta to the sternum. In our case, because of the small space of the child's anterior mediastinum and the huge mass, we could not place an aortic cross-clamp on the ascending aorta, so deep hypothermic circulatory arrest was performed to obtain a bloodless operative field and perfect visualization of the aortic ostium. Hypothermic circulatory arrest was also used in some other studies (3–5). Regarding cardioplegia solution infusion, similar to other studies (4, 5), we infused cardioplegia solution directly into the coronary ostia. This perfusion method is feasible and robust in the emergency condition. Similar to another researcher's experience (5), we didn't perform cerebral perfusion in the redo surgery, and the patient had no cerebral comorbidities because it is safe if the circulatory arrest time is short enough with deep hypothermia.

Apart from surgical excision, we noticed in the literature that there are some alternative treatments. These methods include a hybrid procedure which includes supra-aortic trunk debranching and Ishimaru zone 0 TEVAR with a single chimney to the brachiocephalic trunk (6), percutaneous mechanical occlude implantation to block the AAPA entries (7), and pseudo-aneurysm closure via percutaneous coil embolization followed by insertion of an Amplatzer vascular plug (8). These methods may not be applicable for the child patient in the present case because there was infection co-existing with the pseudo-aneurysm.

We found that the pseudo-aneurysm was raised at the aortic cannulation site confirmed by the evidence that the suture line was found at the ostium. We inferred that the bacteria were planted at the cannulation site due to bacteremia, and the suture lines hindered the antibiotic effects at the cannulation site, which might lead to the development of pseudo-aneurysm.

In summary, the present case describes a serious complication of pseudo-aneurysm at the aortic cannulation site after VSD repairing, and it probably resulted from inappropriate suture and local/systematic infection. The key elements for procedural success were CPB establishment through femoral artery-vein bypass, and the lesions of the aortic wall must be thoroughly cleaned (Supplementary Materials).

It is recommended that our cardiac surgeons shall take extra precautions and monitor infection during the perioperative period when the aortic purse-string suture is made.

## Data Availability Statement

The original contributions presented in the study are included in the article/Supplementary Material, further inquiries can be directed to the corresponding author/s.

## Ethics Statement

The guardian of the patient has given the written informed consent for the publication of this case report.

## Author Contributions

XL and HZ made the study design and data collection, drafted the initial manuscript, and approved the final manuscript as submitted. RZ and JG were part of the surgical team and made data collection, analysis, and interpretation. JZ processed the clinical images including three-dimensional reconstruction of the CT images. TL and JG reviewed and revised the manuscript and approved the final manuscript as submitted. YZ revised the whole manuscript after revision, participated the revision of the manuscription, polishing of the language, and proposed many useful suggestions. All authors contributed to the article and approved the submitted version.

## Conflict of Interest

The authors declare that the research was conducted in the absence of any commercial or financial relationships that could be construed as a potential conflict of interest.

## Abbreviations

VSD: ventricular septal defect
CTA: computed tomograph angiography.
