# Supplementary material for: Case Report: Ascending Aortic Pseudo-Aneurysm Following Ventricular Septal Defect Repair in a 4-Year-Old Girl
Source: Front Pediatr. 2021 Feb 15;9:576527. doi: 10.3389/fped.2021.576527 (PMC7917069; doi:10.3389/fped.2021.576527)
Supplement: Supplementary file 1 [file Data_Sheet_1.pdf]

Patient Perspective

BACKGROUND

I am the child patent's guardian; my kid received VSD repairing operation when she was 4 years old. Everything seems go well after the first surgery, and we discharged at 10 days after operation. But the girl got a high fever (40 degree) a few days later, so we went back to the first affiliated hospital of USTC for further therapy.

SIGNS AND SYMPTOMS/PRESENTATION

The first sign before readmission of my kid is high fever without vomiting and coughing, and it was difficult to get the body temperature back to normal. And the girl's breathing was difficult.

DIAGNOSIS

After the high fever was developed, no definite diagnosis was made until the CTA examination wad done. When doctor told the diagnosis was aortic pseudo-aneurysm, I noticed that it must be very dangerous.

TREATMENTS

Doctors told me that a redo-operation was needed to removal the aneurysm and repair the aorta, and I noticed that there was no better choice beyond that.

COMPLICATIONS

Fortunately, after the redo-operation and ICU therapy, the girl recovered without no special complication eventually.

FOLLOW-UP

The patient now lives in her hometown, and the health condition is well. And we received periodic re-examination at outpatient department until now.
